# Predicting evolutionary responses to selection on polyandry in the wild: additive genetic covariances with female extra-pair reproduction

**DOI:** 10.1098/rspb.2012.1835

**Published:** 2012-09-19

**Authors:** Jane M. Reid

**Affiliations:** Institute of Biological and Environmental Sciences, School of Biological Sciences, Zoology Building, University of Aberdeen, Tillydrone Avenue, Aberdeen AB24 2TZ, UK

**Keywords:** additive genetic covariance, animal model, extra-pair reproduction, polyandry, secondary theorem of selection

## Abstract

The evolutionary forces that underlie polyandry, including extra-pair reproduction (EPR) by socially monogamous females, remain unclear. Selection on EPR and resulting evolution have rarely been explicitly estimated or predicted in wild populations, and evolutionary predictions are vulnerable to bias due to environmental covariances and correlated selection through unmeasured traits. However, evolutionary responses to (correlated) selection on any trait can be directly predicted as additive genetic covariances (cov_A_) with appropriate components of relative fitness. I used comprehensive life-history, paternity and pedigree data from song sparrows (*Melospiza melodia*) to estimate cov_A_ between a female's liability to produce extra-pair offspring and two specific fitness components: relative annual reproductive success (ARS) and survival to recruitment. All three traits showed non-zero additive genetic variance. Estimates of cov_A_ were positive, predicting evolution towards increased EPR, but 95% credible intervals overlapped zero. There was therefore no conclusive prediction of evolutionary change in EPR due to (correlated) selection through female ARS or recruitment. Negative environmental covariance between EPR and ARS would have impeded evolutionary prediction from phenotypic selection differentials. These analyses demonstrate an explicit quantitative genetic approach to predicting evolutionary responses to components of (correlated) selection on EPR that should be unbiased by environmental covariances and unmeasured traits.

## 1. Introduction

Extra-pair reproduction (EPR) by socially monogamous females, and the resulting polyandry, could substantially alter distributions of reproductive success and the evolutionary dynamics of sexually selected traits compared with those arising given strict monogamy [1–3]. The evolutionary forces driving polyandry and EPR are therefore of broad interest but remain far from clear [1,2,4–8]. As for any phenotypic trait, continued evolutionary change (or stasis) in EPR will result from the combined magnitudes of all components of selection coupled with the additive genetic (co)variances underlying EPR and all fitness components and other traits through which selection acts [5,9–11]. Understanding the evolutionary forces driving EPR and polyandry ultimately requires all such selection components and genetic (co)variances to be estimated in populations experiencing natural genetic and environmental variation in reproductive strategy and fitness.

Quantifying the form and magnitude of selection on any trait, and predicting any evolutionary response, is difficult in wild populations where environmental covariation among traits and fitness components may be substantial and all relevant traits, environmental covariates and individuals are unlikely to be measured [11,12–16]. Some relevant parameters can be estimated for single or multiple traits given sufficient data describing variation in phenotype and fitness components among relatives. These include phenotypic selection differentials and gradients with respect to specific fitness components, heritabilities and additive genetic (co)variances [9,10,17,18]. The evolutionary response to selection over one generation attributable to these fitness components can then be predicted using univariate or multivariate breeder's equations [9,10,17].

The approach of estimating phenotypic selection and genetic (co)variances and then predicting consequent evolution is valuable due to its relative empirical tractability and intuitive interpretation, but has limitations [10–13,15]. Widely recognized problems are that phenotypic covariances between focal traits and fitness components, and hence estimated selection differentials, can reflect environmental covariances or indirect effects of other correlated traits rather than direct causal effects. Predicted evolutionary responses can consequently be substantially biased [13–16,19–23].

These problems can in theory be resolved by measuring all relevant environmental covariates and traits, and estimating multivariate (partial) selection gradients with respect to fitness components of interest [9,11,13,14]. However, all such covariates and traits are unlikely to be measured in practice [9,13,15,16,21]. In particular, correlated selection through individuals that die before trait expression, or on reproductive traits through adults that fail to breed, could substantially bias predictions of evolutionary change (creating so-called ‘invisible fractions’ [14,24,25]).

One way to eliminate bias in evolutionary predictions due to environmental covariances and unmeasured traits is to predict responses to selection by directly estimating additive genetic rather than phenotypic (co)variation [13–15,20,21]. Specifically, the predicted evolutionary response to directional selection over one generation, defined as the change in mean breeding value for focal trait *z* (ΔA*_z_*), equals the additive genetic covariance (cov_A_) between the trait and relative fitness (w); ΔA*_z_* = cov_A_(*w*,*z*) (the ‘secondary theorem of selection’ [11,20,26]). Direct estimation of cov_A_(*w*,*z*) can therefore allow unbiased prediction of per-generation evolutionary responses to components of directional selection when environmental variation cannot be controlled or completely measured [14,15,20,21]. Such analyses also allow environmental covariances between traits and fitness components to be explicitly quantified, thereby indicating the degree to which assumptions underlying standard breeder's equation predictions are violated [15,20,21]. An explicit quantitative genetic approach can also allow responses to correlated selection (for example, through individuals that die before trait expression or fail to breed and express reproductive traits) to be predicted because cov_A_ between focal traits and relevant fitness components can be estimated from observed trait values of relatives [14,24].

Despite the potential valuable insights, evolutionary responses to selection have rarely been predicted by estimating cov_A_ between traits and relative fitness components in wild populations, and this approach has not been implemented for female EPR or other forms of polyandry. Even phenotypic selection differentials and gradients on female EPR have not been explicitly estimated with respect to major components of female fitness in the wild. Instead, direct costs and benefits of EPR are typically postulated and estimated in currencies such as decreased paternal care by a female's cuckolded social mate [5] (see §4). No clear consensus regarding such costs and benefits has emerged, not least because observed associations may reflect environmental covariation rather than direct causal effects [27,28]. Explicit estimates of the form, direction and magnitude of components of selection on female EPR in the wild, and consequent evolutionary predictions, therefore remain scant [5,6,8,28].

Estimating additive genetic covariances between female EPR and relative fitness components in wild populations requires appropriate traits to be measured in numerous relatives. I applied ‘animal models’ to 18 years of comprehensive life-history, paternity and pedigree data from song sparrows (*Melospiza melodia*) to estimate cov_A_ between female EPR defined as a female's liability (hereafter ‘EPR_L_’) to produce extra-pair offspring (EPO, sired by an extra-pair male) rather than within-pair offspring (WPO, sired by her socially paired male [29]), and two specific fitness components: adult female annual reproductive success (ARS) and survival to recruitment (SR). These covariances, respectively, predict per-generation evolutionary responses to (correlated) selection on female EPR_L_ associated with female reproductive success, including failure, and pre-reproductive mortality. Evolutionary responses attributable to these two components of selection are of specific interest because they are potentially substantial but cannot be adequately predicted through solely phenotypic analyses. I additionally considered the degree to which evolutionary change in EPR_L_ due to selection through ARS predicted by basic application of the univariate breeder's equation would be biased. I thereby demonstrate a quantitative genetic approach to predicting per-generation evolutionary responses to selection on female liability for EPR as encapsulated by additive genetic covariances with two specific fitness components. Key nomenclature is summarized and defined in table 1.

**Table 1.** Nomenclature.

quantity	definition
EPR_L_	a female's liability to produce an extra-pair offspring (EPO) rather than a within-pair offspring (WPO)
ARS_w_	a female's relative ARS defined as the number of ringed offspring a female produced per year divided by the population mean
SR_w_	an individual's relative SR defined as 1 or 0 for individuals that did or did not survive to age 1 year divided by the population mean; SR_w_ was analysed as a binomial trait (SR_wB_) and as a Gaussian trait (SR_wG_)
V_A_, cov_A_	additive genetic variance and covariance, respectively
V_Y_, cov_Y_	year variance and covariance, respectively
V_PI_, cov_PI_	permanent individual variance and covariance, respectively
V_R_, cov_R_	residual variance and covariance, respectively

## 2. Material and methods

### (a) Study system

Song sparrows of both sexes typically breed two to three times per season starting from age 1 year and are primarily socially monogamous with clear social pairings. Females incubate clutches (typically three to four eggs) and both socially paired parents provision hatched offspring. However, song sparrows are genetically polygynandrous, with frequent EPR [30,31].

Mandarte Island, British Columbia, Canada, (approx. six hectares) holds a resident song sparrow population, recently numbering roughly 15–45 breeding pairs, which has been studied intensively since 1975 [32]. Each year, all breeding attempts are closely monitored and all nests located. All offspring surviving to approximately 6 days post-hatch are marked with unique combinations of coloured rings to allow individual identification. The occasional immigrants to Mandarte (1.1 per year on average) are colour-ringed soon after arrival. All social parents of all offspring (those incubating clutches or provisioning chicks) are identified by observation. Immigration is sufficient to maintain neutral allelic diversity [33] and prevent inbreeding from accumulating.

During 1993–2009, 99.4 per cent of ringed offspring and adults were blood sampled and genotyped at 13 polymorphic microsatellite loci to allow assignment of genetic parents [30]. Offspring were sexed using standard molecular methods [34]. Bayesian full probability models that incorporated genetic and spatial information assigned genetic sires to 99.6 per cent of sampled offspring with at least 95 per cent individual-level confidence. Overall, approximately 29 per cent were assigned to males other than a female's socially paired mate and hence were EPO [30] (compared with 24% in a nearby mainland song sparrow population [31]). The probability of excluding a female's social mate as sire averaged 0.9998, and the number of unsampled sires was effectively zero. All genetic mothers matched those assigned by behaviour [30].

Each adult (at least 1 year old) female's ARS was measured as the number of offspring ringed (approx. 6 days post-hatch) per season. Owing to Mandarte's small size and the intensive fieldwork, the probability of resighting an adult during any breeding season is effectively one. Whether or not each ringed offspring survived to recruit at age one (hereafter ‘SR’) was therefore documented with high confidence [35]. The local recruitment of all ringed offspring and ARS of all adult females in the population were therefore completely described. The high local recruitment rate and scarcity of ringed sparrows on surrounding islands suggest that emigration is relatively rare [32,34]. Since the ‘secondary theorem of selection’ specifies relative rather than absolute fitness [9,20,26], relative ARS and SR (ARS_w_ and SR_w_, table 1) were calculated by dividing individual values by the overall across-year population mean with binary SR coded as 1/0. Estimated additive genetic (co)variances were similar when ARS_w_ and SR_w_ were calculated relative to year-specific rather than across-year means.

### (b) Quantitative genetic analyses

Multivariate ‘animal models’ were used to estimate additive genetic variances (V_A_) and covariances (cov_A_) in and among female EPR_L_, ARS_w_ and SR_w_. Animal models are mixed models in which pairwise coefficients of kinship (*k*) estimated from pedigree data define a matrix proportional to the variance–covariance structure of additive genetic random effects [26,36]. Such models estimate genetic parameters for a baseline population that, in practice, comprises individuals in the pedigree that have unknown parents [36] (see the electronic supplementary material).

Focal traits and fitness components may not be normally distributed, and relative fitness follows no obvious statistical distribution [9,12,22]. While deviation from normality may not bias estimates of additive genetic (co)variances, it does impede hypothesis testing [9,12,17,22]. One solution is to consider traits that take discrete values as ‘threshold traits’ where normally distributed continuous variation in underlying ‘liability’ is assumed to translate into trait expression at certain threshold values [26,37,38]. Statistically appropriate models can then be fitted and interpreted on underlying liability scales. While considering liabilities has statistical advantages, cov_A_ between a trait and liability for relative fitness has no clear quantitative interpretation in terms of predicted evolutionary change. I therefore fitted multiple models (described below) on appropriate observed and liability scales to provide evolutionarily meaningful estimates of cov_A_ and associated statistical confidence.

Female EPR_L_, defined as a female's liability to produce EPO rather than WPO, was modelled as a binomial threshold trait with EPO and ARS as numerator and denominator, respectively [29]. Females whose breeding failed (ARS = 0) are consequently uninformative in univariate analyses of EPR_L_. EPR_L_, defined as a liability rather than simply the observed ratio of EPO to ARS, is not necessarily correlated with ARS at either phenotypic or genetic levels. ARS_w_ was best approximated by a Gaussian distribution (see §3). SR_w_ was modelled as a binary threshold trait (SR_wB_) to estimate cov_A_ with EPR_L_ with interpretable statistical confidence, and as a Gaussian trait (SR_wG_) to provide a quantitatively interpretable evolutionary prediction.

Additive genetic variance in traits and fitness components may be small, especially relative to phenotypic variance. This situation is directly informative since cov_A_ (and hence predicted evolutionary responses to selection) must be zero if V_A_ = 0 [16,20]. However, small V_A_ can impede precise estimation of cov_A_, especially given the limited sample sizes typically available from wild populations [9,15,22]. One approach is to estimate V_A_ in each trait and fitness component of interest and then estimate cov_A_ among traits and components where V_A_ > 0 [15]. In contrast, I fitted pairwise bivariate models between EPR_L_, ARS_w_ and SR_w_ in order to maximize efficient use of available information to estimate V_A_ in case cov_A_ ≠ 0, and hence maximize statistical power [14].

Models included random effects of breeding year for EPR_L_ and ARS_w_, or natal year for SR_w_, and hence estimated among-year variances (V_Y_, table 1) [29,35]. Models for female EPR_L_ and ARS_w_ included random individual effects to account for repeat observations of females that survived multiple years and estimate ‘permanent individual’ variance (V_PI_, which comprises permanent environmental and non-additive genetic variances [36]). These models also included fixed effects of three age categories defined through preliminary analysis (ages 1, 2–3 and greater than 3 years). All models included fixed regressions on individual coefficient of inbreeding (*f*) because unmodelled inbreeding depression may inflate estimates of V_A_, cov_A_ and phenotypic selection [39,40]. The inter-sex genetic correlation for SR is close to one in the study population [35]. Models for SR_w_ therefore used recruitment data from all ringed offspring to maximize statistical power, and included fixed effects of sex because mean SR differs between males and females [34]. Effects were similar when estimated using SR_w_ data for female offspring only. Initial models included random effects of an individual's mother, social father or breeding territory, but estimated variances were close to zero and other parameter estimates were scarcely affected when these terms were removed. Random natal brood effects were not modelled because few same-brood females recruited, and there is little among-brood variance in SR [35].

Model covariance structures were unconstrained, allowing estimation of additive genetic (cov_A_) and year (cov_Y_) covariances among all traits, and of permanent individual (cov_PI_) and residual (cov_R_) covariances between female EPR_L_ and ARS_w_. Since residual variance (V_R_) cannot be estimated and V_PI_ is not identifiable for single-observation binary traits (such as SR_wB_), V_R_ and cov_R_ for SR_wB_ were fixed to one (by convention) and zero, respectively, and V_PI_ and cov_PI_ were not estimated. Cov_R_ between SR_wG_ and EPR_L_ was also fixed to zero because there is zero phenotypic variation in SR_w_ across females whose EPR was observed.

### (c) Analysis implementation

Analyses used SR_w_ data for all offspring ringed during 1993–2009 (when paternity was verified), and EPR_L_ and ARS_w_ for all Mandarte-hatched adult females alive during 1993–2009 (including cases of ARS_w_ = 0). Pedigree data for females and offspring ringed during 1993–2009 were compiled from genetic parentage data taking the most likely sires [29,30,41]. Pedigree data for females and ancestors ringed during 1975–1992 were compiled from observed social parentage and combined with the 1993–2009 genetic data to provide a full pedigree for 1975–2009. Assuming the unobserved EPR rate during 1975–1992 was similar to the approximately 29 per cent observed during 1993–2009 and that all mothers were correctly assigned by social behaviour, approximately 86 per cent of all 1975–1992 pedigree links will be correct. Paternity error for breeders hatched before 1993 will actually be less than this if, as during 1993–2009, EPO were less likely to recruit than WPO [34,35]. The 1975–1992 pedigree data, even though uncorrected for EPR, therefore provide information regarding *k* between the 1993 breeders and allow the alternative assumption of *k* = 0 to be relaxed [41]. Since the contribution of a common ancestor to *k* between two focal individuals decreases by a factor of two per generation of separation, paternity error in the early pedigree introduces little error into estimates of *k* among recent breeders. Using the full 1975–2009 pedigree data therefore allows full use of all available phenotypic data and provides the most powerful analysis feasible (see [29,41]). Animal model pedigrees were pruned to individuals with phenotypic data and all their known ancestors, and thereby restricted to individuals that are informative for current analyses (see the electronic supplementary material). Individual *f*-values were calculated from the 1975–2009 pedigree using standard algorithms, and therefore measure the probability of identity by descent relative to pedigree founders [42]. Kinship between immigrants and existing natives, and hence *f* of offspring of immigrant–native pairings, was defined as zero [42,43]. Phenotypic data from 11 immigrant females were excluded because *f* is undefined for immigrants (as opposed to their offspring).

Models were fitted using Bayesian methods implemented in MCMCglmm 2.14 in R v. 2.12.2 [44,45] with logit link functions for threshold traits. Fixed effect priors were normally distributed and diffuse with mean zero and large variance (10^8^). Prior variances were inverse-Wishart distributed with limit variance of one and low degree of belief (0.002). Prior covariances were zero with low degree of belief (0.002). Priors were therefore only weakly informative, and posterior distributions were robust to reasonable prior variation, including covariances of −0.5 to 0.5. Analyses used at least 3 005 000 iterations, burn-in 5000 and thinning interval 3000 to ensure low (less than 0.05) autocorrelation among thinned samples. Posterior means and 95 per cent credible intervals (95% CI) for fixed effects, (co)variances and heritabilities (*h*^2^) were estimated across thinned samples. Lower limits on estimates are bounded to zero for variances but not covariances. Posterior distributions for variances were therefore further inspected to assess modes and density close to zero, and hence evaluate the evidence that variances differed from zero. Simulations of null traits with V_A_ = 0 further substantiated these inferences (see the electronic supplementary material). Liability-scale heritabilities of EPR_L_ and SR_wB_ were estimated from binomial models as 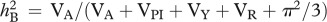 given logistic variance proportional to *π*^2^/3 [46]. Heritabilities of ARS_w_ and SR_wG_ were estimated as 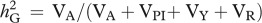. To aid interpretation, the per-generation magnitude of evolutionary change in EPR attributable to selection through ARS_w_ or RS_w_ predicted on the liability scale was inverse-logit back-transformed to the observed phenotypic scale.

Full trivariate models for EPR_L_, ARS_w_ and SR_w_ could not be properly fitted in MCMCglmm because the correct residual covariance structure (with estimated cov_R_ between EPR_L_ and ARS_w_ but zero cov_R_ with SR_w_) cannot be specified. A further bivariate model was therefore fitted to estimate cov_A_ between ARS_w_ and SR_wB_, and hence consider whether estimates of cov_A_ between EPR_L_ and ARS_w_ or SR_wB_ could be biased by unmodelled cov_A_ with SR_wB_ or ARS_w_, respectively. Trivariate models fitted as rigorously as feasible supported conclusions drawn from bivariate models (see the electronic supplementary material). Data are available at the Dryad Repository: doi:10.5061/dryad.907cv.

### (d) Phenotypic selection differential

My primary aim was to estimate cov_A_ between EPR_L_ and two specific components of relative fitness (ARS_w_ and SR_w_). However, I also compared resulting evolutionary predictions with basic implementation of the univariate breeder's equation, *R* = *h*^2^*S*, where the phenotypic selection differential (*S*) equals the phenotypic covariance (cov_P_) between trait and relative fitness, *S* = cov_P_(*z*,*w*) [9,15]. Cov_P_ between EPR_L_ and ARS_w_ was estimated as the residual covariance from a bivariate mixed model (assuming binomial and Gaussian error distributions, respectively). This model included random year effects and fixed effects of age and *f* (as in the animal model used to estimate cov_A_), but estimates remained similar when these fixed effects were excluded. Cov_P_ was therefore estimated across female-years where ARS_w_ exceeded zero, and hence from a subset of the data used to estimate cov_A_ (see §3). Cov_P_ between EPR_L_ and SR_w_ is zero because EPR is only observable for females that recruited.

## 3. Results

### (a) Female extra-pair reproduction and annual reproductive success

**Figure 1.**
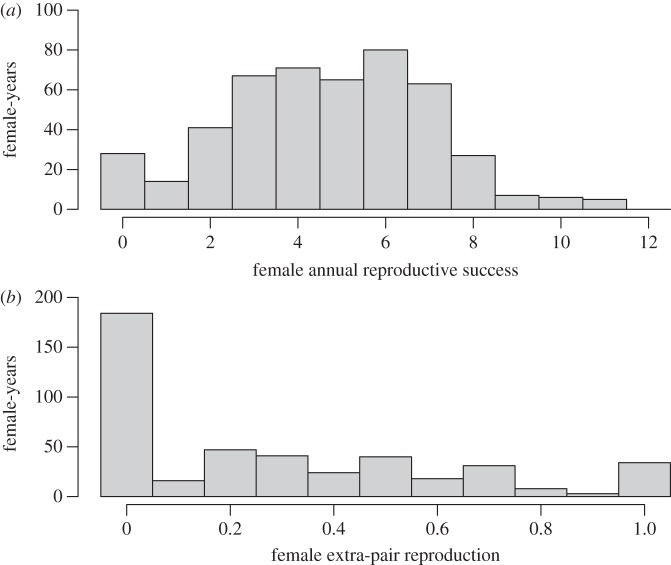
Distributions of female (*a*) annual reproductive success (ARS, the number of offspring ringed per year) and (*b*) annual EPR (visualized as the observed proportion of ringed offspring that was sired by an extra-pair male) across adult female song sparrows alive during 1993–2009.

A total of 224 Mandarte-hatched adult female song sparrows were alive during 1993–2009, totalling 474 female-years (see the electronic supplementary material). Female ARS varied from zero to 11 ringed offspring (mean 4.7, variance 5.5, median 5, inter-quartile range 3–6, skew −0.1; figure 1). The distribution of ARS_w_ differed from normality (Kolmogorov–Smirnov *p* = 0.005) but was only slightly skewed. Overall, 211 of the 224 females had at least one offspring in at least one year during 1993–2009, totalling 446 female-years. Across these cases, the observed proportion of ringed offspring that were EPO ranged from zero to one (mean 0.29, variance 0.10, median 0.20, skew 0.83; figure 1). This proportion did not increase or decrease during 1993–2009 (correlation coefficient *r* = 0.001).

The pedigree comprising the 224 females and all their known ancestors totalled 479 individuals (see the electronic supplementary material). Mean pairwise *k* was 0.055 among all 479 individuals (median 0.051, inter-quartile range 0.020–0.075, range 0.000–0.471, 9.0% zeros) and 0.072 among the 224 females whose ARS was observed (median 0.065, inter-quartile range 0.049–0.084, range 0.000–0.409, 0.7% zeros). Mean *f* was 0.061 across these females (median 0.053, inter-quartile range 0.031–0.083, range 0.00–0.305).

### (b) (Co)variances in female EPR_L_ and ARS_w_

The bivariate animal model for female EPR_L_ and ARS_w_ estimated non-zero V_A_ and *h*^2^ in both traits with low posterior density close to zero, and significant inbreeding depression in ARS_w_ but not EPR_L_ (table 2). Estimated cov_A_ between EPR_L_ and ARS_w_ was positive but small, and the 95% CI overlapped zero (table 2). Estimates of V_PI_ in EPR_L_ and ARS_w_ were small with relatively high posterior density towards zero, and cov_PI_ was small with a 95% CI that overlapped zero (table 2). There was substantial V_Y_ in ARS_w_ but not EPR_L_, and consequently small cov_Y_ with a 95% CI that overlapped zero (table 2). There was substantial V_R_ in both EPR_L_ and ARS_w_, and negative cov_R_ with a 95% CI that did not overlap zero (table 2).

**Table 2.** Posterior means (and 95% credible intervals) for additive genetic, permanent individual, year and residual (co)variances between female liability to produce an extra-pair offspring (EPR_L_), relative annual reproductive success (ARS_w_) and relative SR on liability and observed scales (SR_wB_ and SR_wG_), and heritability and inbreeding depression, estimated from bivariate animal models. Heritabilities were calculated on Gaussian (*h*_G_^2^) or binomial (*h*_B_^2^) scales. Note that 95% CIs for V_A_ and *h*^2^ do not converge to zero.

	additive genetic variance (V_A_)	permanent individual variance (V_PI_)	year variance (V_Y_)	residual variance (V_R_)	heritability (*h*^2^)	inbreeding depression (*β*_*f*_)	additive genetic covariance (cov_A_)	permanent individual covariance (cov_PI_)	year covariance (cov_Y_)	residual covariance (cov_R_)
ARS_w_	0.016 (0.002–0.039)	0.022 (<0.0001–0.026)	0.060 (0.020–0.120)	0.147 (0.121–0.172)	*h*^2^_G_: 0.068 (0.002–0.154)	−1.07 (−2.06–−0.08)	0.087 (−0.027–0.199)	0.018 (−0.070–0.117)	−0.011 (−0.082–0.050)	−0.105 (−0.205–−0.007)
EPR_L_	1.198 (0.226–271)	0.139 (<0.0001–0.627)	0.024 (0.001–0.086)	2.015 (1.302–2.788)	*h*^2^_B_: 0.177 (0.052–0.311)	−0.17 (−6.75–6.34)				
SR_wB_	0.405 (0.066–0.768)	—	0.744 (0.202–1.483)	1 (fixed)	*h*^2^_B_: 0.073 (0.014–0.133)	−8.04 (−11.80–−4.28)	0.160 (−0.188–0.522)	—	0.036 (−0.216–0.295)	0 (fixed)
EPR_L_	1.217 (0.497–2.093)	0.210 (<0.0001–0.444)	0.028 (0.0002–0.091)	1.910 (1.263–2.611)	*h*^2^_B_: 0.182 (0.090–0.297)	−1.59 (−8.36–4.51)				
SR_wG_	0.154 (0.035–0.278)	—	0.297 (0.070–0.579)	3.822 (3.601–4.080)	*h*^2^_B_: 0.036 (0.010–0.064)	−4.50 (−6.33–−2.57)	0.093 (−0.113–0.320)	—	0.018 (−0.139–0.201)	0 (fixed)
EPR_L_	1.002 (0.013–1.810)	0.174 (<0.0001–0.862)	0.030 (0.0003–0.106)	1.978 (1.241–2.699)	*h*^2^_B_: 0.152 (0.006–0.256)	−1.56 (−8.10–3.93)				
SR_wB_	0.398 (0.073–0.796)	—	0.750 (0.174–1.526)	1 (fixed)	*h*^2^_B_: 0.072 (0.015–0.127)	−8.03 (−11.55–−4.32)	0.025 (−0.047–0.090)	—	0.033 (−0.120–0.166)	0 (fixed)
ARS_w_	0.015 (0.001–0.038)	0.019 (<0.001–0.045)	0.054 (0.016–0.108)	0.154 (0.127–0.179)	*h*^2^_G_: 0.062 (0.002–0.158)	−1.24 (−2.28–−0.15)				

Across 446 observations of 211 females with non-zero ARS, the phenotypic covariance (cov_P_) between EPR_L_ and ARS_w_ was –0.026 (95% CI –0.117–0.075).

### (c) Survival to recruitment

Of 2329 offspring ringed during 1993–2009, 453 (19.5%) survived to recruit (age 1 year). The pedigree comprising all females and offspring and all their known ancestors totalled 2568 individuals (see the electronic supplementary material). Mean pairwise *k* was 0.066 among all 2568 individuals (median 0.062, inter-quartile range 0.045–0.080, range 0.000–0.471, 1.5% zeros) and 0.070 among the 2369 individual females and offspring that contributed phenotypic data (median 0.064, inter-quartile range 0.050–0.082, range 0.000–0.421, 0.1% zeros). Mean *f* was 0.071 across all 2329 offspring (median 0.066, inter-quartile range 0.040–0.092, range 0.000–0.305).

### (d) (Co)variances in female EPR_L_, ARS_w_ and SR_w_

The bivariate animal model for female EPR_L_ and SR_wB_ estimated non-zero V_A_ and *h*^2^ in both traits with low posterior density close to zero, and substantial inbreeding depression in SR_wB_, but not EPR_L_ (table 2). Estimated cov_A_ between EPR_L_ and SR_wB_ was positive, but the 95% CI was wide and overlapped zero (table 2). There was substantial V_Y_ in SR_wB_, but small cov_Y_ with a 95% CI that overlapped zero (table 2). Estimated cov_A_ between EPR_L_ and SR_wG_ was also positive but small (table 2).

The bivariate animal model for ARS_w_ and SR_wB_ estimated that cov_A_ between these fitness components was small with a narrow 95% CI that overlapped zero (table 2).

## 4. Discussion

Despite huge interest in identifying forces driving the evolution of polyandry and female EPR [1,2,4,8], selection differentials or gradients on EPR and corresponding evolutionary responses have not been explicitly estimated or predicted in wild populations [5,6]. Direct costs and benefits of EPR have instead been postulated in terms of decreased paternal care by a female's cuckolded social mate, time, energy or disease costs of mating, or increased fertility or foraging opportunities [1,5,8,47]. However, estimating such effects is difficult because female EPR cannot be readily manipulated in isolation from other behavioural or life-history traits of a female and/or her social mate [47]. Without such experiments, estimated costs and benefits may be biased by environmental covariances between EPR and behaviour, physiology or life history [8,27,28], and may inadequately represent fitness anyway.

Direct selection on polyandry has been experimentally demonstrated in laboratory populations of facultatively polyandrous taxa, particularly where males provide nuptial resources or sperm is limiting, and female fecundity consequently increases with multiple mating and polyandry *per se* [8] (but see [2]). In contrast, the hypothesis that EPR by socially monogamous females is under positive direct selection through increased female fecundity or reproductive success is often rejected because there is no obvious mechanism rather than through rigorous empirical test. Female EPR is consequently hypothesized to be under positive indirect selection through increased offspring fitness [1,4,28]. However, tests of this hypothesis, which typically quantify whether EPO are phenotypically fitter than their maternal half-sib WPO, have themselves failed to demonstrate any consistent positive effect and may be biased anyway [5,8,34,35,48]. Furthermore, phenotypic selection analyses cannot predict evolutionary responses to correlated selection on female EPR through pre-reproductive mortality or reproductive failure unless all correlated traits are identified and measured [14,24,25]. Purely phenotypic approaches may consequently be inadequate to accurately predict evolutionary responses to (correlated) selection on EPR and polyandry through major fitness components, such as recruitment and reproductive success.

To attempt to circumvent these problems, I estimated additive genetic covariances (cov_A_) between female liability to produce EPO (EPR_L_) and relative annual reproductive success (ARS_w_) and survival to recruitment (SR_w_) in song sparrows, thereby parametrizing the ‘secondary theorem of selection’ with respect to these two specific fitness components. I thereby demonstrate an approach to predicting per-generation evolutionary responses associated with specific components of (correlated) selection on female EPR_L_ that should be unbiased by environmental covariances and unmeasured traits.

### (a) Genetic (co)variances

There was moderate additive genetic variance (V_A_) and heritability (*h*^2^) in female EPR_L_ in song sparrows (see also [29]), and small but non-zero V_A_ and *h*^2^ in female ARS_w_. There was therefore potential for non-zero cov_A_ between EPR_L_ and ARS_w_, and hence for a positive or negative evolutionary response to selection on EPR_L_ through female ARS. In fact, estimated cov_A_ between EPR_L_ and ARS_w_ was positive, predicting evolution towards increased female EPR_L_. The posterior mean estimate of approximately 0.09 (table 2) equates to a predicted increase in the proportion of offspring that are EPO of approximately 0.02 per generation on the back-transformed observed scale. However, although the 95% CI was reasonably narrow (−0.03 to 0.20), reflecting the completeness of the song sparrow pedigree and the relatively high relatedness among observed females, it overlapped zero. There was therefore no conclusive prediction of an evolutionary increase in female EPR_L_ associated with selection through female ARS. This analysis incorporates correlated selection through reproductive failure (ARS = 0), which cannot contribute to direct selection on EPR_L_ defined as a female's liability to produce EPO rather than WPO or hence be detected by univariate phenotypic selection analysis.

There was also significant V_A_ and *h*^2^ in SR in song sparrows (see also [35]) and hence potential for non-zero cov_A_ between EPR_L_ and SR_w_. Such covariance, either positive or negative, can be interpreted as a predicted evolutionary response to correlated selection on EPR_L_ through pre-recruitment mortality, and hence through individuals that die before EPR_L_ can be expressed [14,24]. Any such selection is by definition indirect, for example reflecting genetic variation underlying physiology or behaviours that affect both recruitment and EPR_L_. Estimated cov_A_ between EPR_L_ and SR_w_ was positive, implying evolution towards increased female EPR_L_ through correlated viability selection. However, the 95% CI for SR_wB_ was wide (−0.19 to 0.52) despite the relatively large number of observed individuals and overlapped zero. This reflects reduced power to estimate cov_A_ among traits expressed across rather than within individuals, and intrinsic sampling variance associated with estimating liabilities underlying binary traits such as recruitment. There was therefore no conclusive prediction of evolutionary change in female EPR_L_ owing to correlated selection through pre-reproductive mortality.

Estimated cov_A_ between ARS_w_ and SR_wB_ was small with a narrow 95% CI that overlapped zero (−0.05 to 0.09). There was therefore no evidence of a substantial genetic trade-off between liability to recruit and female ARS as might be most simply manifested as negative cov_A_ [49] (but see [50]). Estimated cov_A_ values between EPR_L_ and ARS_w_ and SR_w_ estimated from bivariate models are therefore unlikely to be biased by unmodelled cov_A_ with SR_w_ and ARS_w_, respectively. Indeed, estimates of cov_A_ from trivariate models were qualitatively similar to those from pairwise bivariate models and were therefore robust across the set of three traits considered (see the electronic supplementary material).

Polyandrous female song sparrows were previously shown to produce EPO of lower phenotypic and additive genetic value for fitness components than their WPO [34,35]. There may therefore be weak negative selection against female EPR_L_ owing to low additive genetic value of EPO [35]. In contrast, estimates of cov_A_ between EPR_L_ and ARS_w_ and SR_w_ were positive (although not significantly different from zero). Since combined selection on any trait equals the sum of selection through multiplicative components of relative fitness [10], the response to selection on EPR_L_ associated with SR and subsequent ARS can be predicted as the sum of cov_A_ with SR_wG_ and ARS_w_, giving roughly 0.18 (table 2). This equates to a predicted evolutionary increase in the proportion of offspring that are EPO of approximately 0.03 per generation on the back-transformed observed scale associated with these two specific fitness components. Combined positive selection on female EPR_L_ associated with ARS and SR may therefore partially balance negative selection through low additive genetic value of EPO and help maintain EPR in this population. However, an appropriate 95% CI for the summed cov_A_ is hard to estimate because SR_w_ is not normally distributed, and there is no clear quantitative evolutionary interpretation of cov_A_ between EPR_L_ and liability for relative recruitment.

### (b) Environmental covariances

Joint estimation of additive genetic and environmental covariances between phenotypic traits and relative fitness components, as achieved by fitting ‘animal models’, allows evolutionary responses to selection to be predicted independent of environmental covariances that can confound breeder's equation predictions [15]. Estimating both these distinct covariances provides biological insight and allows key assumptions underlying breeder's equation predictions to be validated [15,20,21].

In song sparrows, the ‘permanent individual’ covariance (cov_PI_) between female EPR_L_ and ARS_w_, which comprises permanent environmental and non-additive genetic effects that consistently influence individual values of both traits, was positive but small. However, the residual covariance (cov_R_), which comprises season-specific individual effects on both traits, was significantly negative, and hence opposite in sign to the posterior mean cov_A_. Negative cov_R_ implies that females with high ARS_w_ due to season-specific individual environmental effects also have low EPR_L_. This could, for example, reflect correlated environmental effects on female ARS_w_ and male mate guarding (if both vary with resource availability), or negative effects of female EPR on paternal care and hence ARS_w_, or other similar mechanisms [47].

The estimated phenotypic covariance (cov_P_) between female EPR_L_ and ARS_w_ was negative (although not significantly different from zero), reflecting the negative cov_R_. The evolutionary response to selection on EPR_L_ predicted by simple application of the univariate breeder's equation (*R* = *h*^2^*S* ≈ 0.17 × −0.03 ≈ −0.005) would consequently be opposite in sign to the posterior mean cov_A_ (contra [21]). The long-standing concern that phenotypic associations between EPR and fitness components may partly reflect environmental covariances is therefore justified [27,28]. However, the 95% CI for cov_A_ between ARS_w_ and EPR_L_ of −0.027 to 0.199 includes the point estimate of *R* from the univariate breeder's equation. Bias in predicted evolutionary change was consequently relatively small in the current case.

### (c) Context and interpretation

Estimating selection on any trait through any fitness component(s), and predicting any consequent evolutionary response, is extremely challenging in wild populations [11,13,21]. Any association-based estimates, whether phenotypic or quantitative genetic, should be interpreted cautiously, and ideally confirmed experimentally [12,16,20,25]. However, for traits such as EPR that cannot be readily experimentally manipulated in the wild, quantitative genetic approaches have advantages over purely phenotypic analyses; namely that evolutionary responses to (correlated) selection can be predicted without bias owing to environmental covariances or unmeasured traits [14,15,20,24]. The current analyses demonstrate this approach with respect to EPR_L_ and two specific fitness components, but some provisos and challenges of application and interpretation remain.

Evolutionary change in female EPR_L_ will ultimately depend on cov_A_ with all components of female and male lifetime fitness from conception [11], including relative adult survival, male ARS (given that cross-sex genetic correlations for fitness components may be less than one [51]) and survival to hatch as well as ARS_w_ and SR_w_. Indirect genetic effects stemming from male–female interactions, correlated selection through associated mating behaviours (such as extra-pair copulations rather than EPR *per se*) and non-additive genetic effects could also contribute [6,20,52,53]. Substantial further theory, data and analyses are required to estimate and combine all possible components of selection on EPR_L_, and achieve the ultimate goal of predicting and understanding the evolution of polyandry. The most tractable and insightful approach to this task is initially to quantify and understand each selection component individually. Accordingly, my current aim was to estimate cov_A_ between EPR_L_ and two specific fitness components (ARS_w_ and SR_w_), and hence predict per-generation evolutionary responses to selection associated with these two specific components. Failure to model further genetically correlated traits and fitness components does not invalidate conclusions regarding the two focal components, but does impede clear distinction between predicted responses to direct and indirect (correlated) selection [20], and means that overall evolutionary responses cannot yet be predicted.

Furthermore, the basic secondary theorem of selection predicts the evolutionary response to directional selection over one generation. In general, ‘animal model’ analyses that use phenotypic data from multiple (overlapping) generations provide unbiased estimates of key genetic parameters in the baseline population [26,36] (see the electronic supplementary material). Such analyses therefore do not necessarily measure or explain contemporary evolutionary or phenotypic dynamics of EPR_L_, but rather provide point predictions of evolutionary responses associated with specific components of selection. Moreover, the secondary theorem of selection predicts the evolutionary response to directional selection in terms of a change in mean breeding value for the focal trait (ΔA*_z_*). This approach can be extended to estimate the response to stabilizing or disruptive selection, defined as a change in additive genetic variance (ΔV_A*z*_), as cov_A_ between relative fitness and the squared deviation of the trait from its phenotypic mean (ΔV_A*z*_ = cov_A_(*w*,(*z* − *μ*_*z*_)^2^), where *μ*_*z*_ is mean *z* [20,23]). However, this covariance cannot be readily estimated for liabilities underlying threshold traits (such as EPR_L_), meaning that potential responses to nonlinear selection are not easily predictable.

Low power is likely to afflict most field estimates of genetic covariances, particularly those involving fitness components [16,21,22]. The song sparrow dataset provides reasonable power for such analyses despite the relatively small number of observed individuals; the comprehensive pedigree data mean that relatedness between these individuals is estimated relatively accurately and is typically non-zero. Genetic covariances of roughly 0.15–0.25 can consequently be detected [35]. However, even given this dataset, the 95% CIs for the estimated cov_A_ between EPR_L_ and SR_wB_ were wide, indicating low power stemming from high sampling variance associated with estimating liabilities underlying threshold traits. The most robust general conclusions regarding components of selection on EPR, and other traits, may ultimately come through meta-analysis [2,8], if sufficient unbiased estimates of key parameters can be provided. Cov_A_ between EPR_L_ and relative fitness components should therefore be estimated as precisely as feasible in a range of populations. This should become increasingly tractable as multi-generational paternity assignment studies that encompass large sample sizes become available.
